# On the difficulties of characterizing weak protein interactions that are critical for neurotransmitter release

**DOI:** 10.1002/2211-5463.13473

**Published:** 2022-09-02

**Authors:** Josep Rizo, Guillaume David, Michael E. Fealey, Klaudia Jaczynska

**Affiliations:** ^1^ Department of Biophysics University of Texas Southwestern Medical Center Dallas TX USA; ^2^ Department of Biochemistry University of Texas Southwestern Medical Center Dallas TX USA; ^3^ Department of Pharmacology University of Texas Southwestern Medical Center Dallas TX USA

**Keywords:** complexin, membrane fusion, neurotransmitter release, SNAREs, synaptotagmin, weak protein interactions

## Abstract

The mechanism of neurotransmitter release has been extensively characterized, showing that vesicle fusion is mediated by the SNARE complex formed by syntaxin‐1, SNAP‐25 and synaptobrevin. This complex is disassembled by *N*‐ethylmaleimide sensitive factor (NSF) and SNAPs to recycle the SNAREs, whereas Munc18‐1 and Munc13s organize SNARE complex assembly in an NSF‐SNAP‐resistant manner. Synaptotagmin‐1 acts as the Ca^2+^ sensor that triggers exocytosis in a tight interplay with the SNAREs and complexins. Here, we review technical aspects associated with investigation of protein interactions underlying these steps, which is hindered because the release machinery is assembled between two membranes and is highly dynamic. Moreover, weak interactions, which are difficult to characterize, play key roles in neurotransmitter release, for instance by lowering energy barriers that need to be overcome in this highly regulated process. We illustrate the crucial role that structural biology has played in uncovering mechanisms underlying neurotransmitter release, but also discuss the importance of considering the limitations of the techniques used, including lessons learned from research in our lab and others. In particular, we emphasize: (a) the promiscuity of some protein sequences, including membrane‐binding regions that can mediate irrelevant interactions with proteins in the absence of their native targets; (b) the need to ensure that weak interactions observed in crystal structures are biologically relevant; and (c) the limitations of isothermal titration calorimetry to analyze weak interactions. Finally, we stress that even studies that required re‐interpretation often helped to move the field forward by improving our understanding of the system and providing testable hypotheses.

## Graphical abstract

The variety of synaptotagmin‐1‐SNARE interactions that have been described illustrates the challenges involved in determining the protein interactions that control the different steps leading to Ca^2+^‐triggered synaptic vesicle fusion.

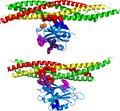

**Abbreviations**
1D: one dimensional
2D: two dimensional
cryo‐EM: cryo‐electron microscopy
cryo‐ET: cryo‐electron tomography
DAG: diacylglycerol
EPR: electron paramagnetic resonance
FRET: fluorescence resonance energy transfer
HOPS: homotypic fusion and vacuole protein sorting
HSQC: heteronuclear single quantum coherence
ITC: isothermal titration calorimetry
MD: molecular dynamics
NMR: nuclear magnetic resonance
NSF: *N*‐ethylmaleimide sensitive factor
PIP_2_
: phosphatidylinositol 4,5‐bisphosphate
PS: phosphatidyl serine
RIM: Rab‐interacting molecule
RRP: readily‐releasable pool
SNAP: soluble NSF attachment protein
SNAP‐25: synaptosomal‐associated protein 27 kDa
SNARE: SNAP receptor
Syt1: synaptotagmin‐1
TM: transmembrane
WT: wild type
ZF: zinc finger

The release of neurotransmitters by Ca^2+^‐evoked synaptic vesicle exocytosis mediates communication between neurons and is thus crucial for brain function. This process involves tethering of synaptic vesicles at specialized areas of the presynaptic plasma membrane called active zones, a priming step(s) that leaves the vesicles ready for release, and fast (< 1 ms) vesicle fusion upon Ca^2+^ influx into a presynaptic terminal [1]. Extensive studies have characterized the protein machinery that exquisitely controls each of these steps (reviewed in Refs [2, 3, 4, 5, 6]), which has allowed reconstitution of basic features of synaptic vesicle fusion with the key components of this machinery [7, 8, 9] and has defined their functions (Fig. 1). These components include the SNARE proteins synaptobrevin (also called VAMP), syntaxin‐1 and SNAP‐25, which form a tight four‐helix bundle called the SNARE complex that brings the two membranes together and is crucial for membrane fusion [10, 11, 12, 13] (Fig. 1C,D). *N*‐ethylmaleimide sensitive factor (NSF) and SNAPs (unrelated to SNAP‐25) disassemble this complex to recycle the SNAREs for another round of fusion [10, 14] (Fig. 1A,D) and to ensure that SNARE complex assembly occurs through a highly regulated, NSF/SNAP‐resistant pathway whereby SNARE complex assembly is organized by Munc18‐1 and Munc13s [7, 15, 16, 17, 18]. This pathway is initiated by binding of Munc18‐1 to a self‐inhibited ‘closed’ conformation of syntaxin‐1 [19, 20] (Fig. 1A). Munc13‐1 bridges the vesicle and plasma membranes [21, 22, 23] and helps open syntaxin‐1 [24, 25, 26, 27] while Munc18‐1 also binds to synaptobrevin [28], forming a template for SNARE complex assembly [29, 30, 31, 32, 33] (Fig. 1B). The resulting partially assembled trans‐SNARE complexes form the core of the primed state [34] (Fig. 1C) and can be disassembled by NSF/SNAPs, but are protected against de‐priming by Munc18‐1 and Munc13‐1 [16, 35, 36]. Synaptotagmin‐1 (Syt1) acts as the major Ca^2+^ sensor that triggers release [37, 38] and functions in a tight interplay with complexins [39, 40, 41] whereby both Syt1 and complexins bind to the trans‐SNARE complexes [42, 43, 44, 45], likely contributing also to protection against disassembly by NSF/SNAPs [16] and keeping the system in a spring‐loaded state that prevents premature fusion but is ready to trigger fast fusion upon Ca^2+^ influx [3] (Fig. 1C,D).

**Fig. 1:**
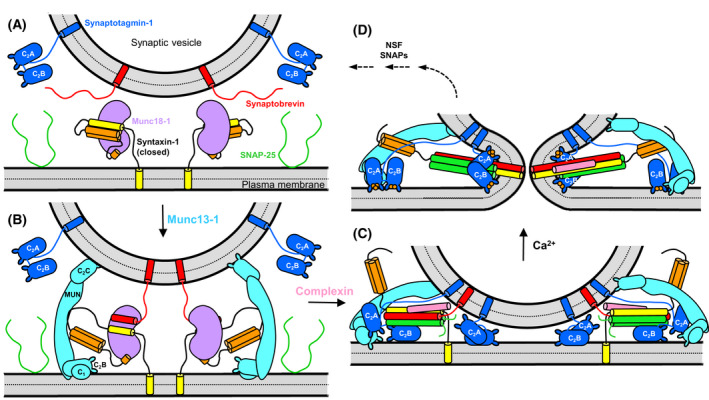
Working model of the steps that lead to neurotransmitter release. (A) Diagram showing the localization of synaptobrevin (red) and Syt1 (blue) on a synaptic vesicle and of SNAP‐25 (green) and syntaxin‐1 (N‐peptide and H_abc_ domain in orange; SNARE motif and TM region in yellow) on the plasma membrane. Syntaxin‐1 adopts a closed conformation in which the SNARE motif binds intramolecularly to the H_abc_ domain. This closed conformations binds tightly to Munc18‐1 (purple) and this complex is the starting point of the pathway that leads to synaptic vesicle fusion. (B) Munc13‐1 (cyan) bridges the vesicle and plasma membranes and binds to the linker joining the H_abc_ domain and the SNARE motif of syntaxin‐1, helping to open syntaxin‐1. Binding of synaptobrevin to Munc18‐1 forms the template complex. (C) Binding of SNAP‐25 to syntaxin‐1 and synaptobrevin leads to partial assembly of the SNARE four‐helix bundle. Syt1 binds to the SNARE complex and the plasma membrane through the C_2_B domain, and complexin (pink) binds to the other side of the SNARE complex, stabilizing the complex and likely preventing dissociation of the SNARE complex by NSF/SNAPs but at the same time hindering premature fusion. (D) Ca^2+^ influx leads to dissociation of Syt1 from the SNARE complex due to tight Ca^2+^‐ and PIP_2_‐dependent binding to the plasma membrane. The same Syt1 molecules or others that might be in the space between the vesicle and the plasma membrane (C) cooperate with the SNARE complex in inducing membrane fusion. In (C, D), the complexin N‐ and C‐terminal regions, as well as the flexible linker joining the SNAP‐25 SNARE motifs, are not shown for simplicity.

Elucidating these molecular mechanisms has been challenging because the release machinery is assembled between two membranes and because by its very nature this system is highly dynamic. Early studies by nuclear magnetic resonance (NMR) spectroscopy and/or X‐ray crystallography determined the three‐dimensional structures of isolated proteins involved in release [46, 47, 48, 49, 50, 51, 52, 53, 54, 55, 56, 57, 58, 59, 60] and of tight complexes between them such as the SNARE complex, the closed syntaxin‐1/Munc18‐1 complex and the complexin‐1/SNARE complex [13, 20, 43] using soluble fragments. Although the structures provided seminal insights, they constitute snapshots of a dynamic, malleable system where multiple energy barriers hinder the transitions that eventually lead to Ca^2+^‐triggered fusion. Moreover, weak interactions (*K*_d_ > 10 μm) among these proteins as well as between the proteins and the lipids can play critical roles by lowering the energy barriers of these transitions. Unfortunately, weak interactions are more difficult to characterize than strong ones, in part because it is more difficult to crystallize the complexes, and in part because they may be masked by spurious interactions that may be stronger than the physiologically relevant interactions. Moreover, weak interactions are more likely to be dependent on molecular crowding effects that occur in cells and are difficult to reproduce in studies performed *in vitro*. Weak binding between soluble proteins can be strengthened by co‐localization in the restricted volume of the system and by cooperativity with protein‐lipid interactions in the presence of membranes, yielding metastable states that may represent key intermediates in the pathway to fusion. Inclusion of membranes can facilitate the formation of the relevant complexes but hinders crystallization and application of NMR spectroscopy [61]. Use of these techniques is even more difficult if complexes need to be assembled between two membranes. Cryo‐electron microscopy (Cryo‐EM) has emerged as a powerful tool to determine the structure of membrane‐anchored complexes, but weak complexes are also difficult to study by cryo‐EM, and cryo‐EM analyses of Syt1, the SNAREs and complexin present additional challenges because of their small size and the abundance of flexible regions.

Research on neurotransmitter release has provided a vivid illustration of these problems and of how they sometimes can be overcome with creativity, innovation and, particularly, persistence. At the same time, there are also examples of studies that had to be re‐interpreted because, initially, some technical limitations of the supporting experiments were not sufficiently recognized. For instance, there is a polybasic sequence of Syt1 that binds to membranes but is highly promiscuous and confounded many studies of Syt1 interactions performed in solution in the absence of membranes, including some studies from our own laboratory (please note that in this review we use the terms polybasic and poliacidic, which are widely employed in the protein literature, to refer to the more accurate terms polycationic and polyanionic, respectively). There are also cases where the fact that protein crystallization necessarily favors weak protein–protein interactions that form the crystal lattice, and most often are irrelevant, was not sufficiently considered. Some isothermal titration calorimetry (ITC) studies of weak interactions have also required re‐interpretation because of unexpected contributions from nonspecific interactions to the observed heat. We stress that these issues are natural in research of such a complicated biological process and that studies that required re‐interpretation often yielded useful information that helped to make progress in the field.

The purpose of this review is to discuss technical aspects that illustrate the complexities involved in studying neurotransmitter release and lessons learned from these aspects, while at the same time describing the protein interactions that underlie the steps leading to release outlined above. We also aim to clarify which mechanistic concepts in this area are well established and which need to be tested further. We start with studies of the SNAREs, complexins and Syt1 that were rich in technical difficulties and illuminated the last stages of the release mechanism. Later we discuss interactions that underlie how Munc18‐1 and Munc13‐1 orchestrate SNARE complex assembly in earlier stages that lead to synaptic vesicle priming. We hope that the review will be of interest not only to scientists in this field but also more generally to those who investigate molecular mechanisms underlying biological processes.

## SNARE complexes

**Fig. 2:**
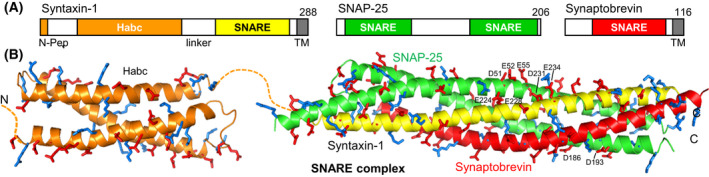
The SNARE complex. (A) Domain diagram of syntaxin‐1, SNAP‐25 and synaptobrevin with the length of each protein indicated by the number on the right, above each diagram. SNARE indicates SNARE motif. N‐pep, N‐peptide. (B) Ribbon diagrams of the NMR structure of the syntaxin‐1 H_abc_ domain (orange) [48] (PDB accession code 1BR0) and the SNARE four‐helix bundle [13] (PDB accession code 1SFC) with syntaxin‐1 SNARE motif in yellow, SNAP‐25 in green and synaptobrevin in red. Acidic and basic side chains are shown as stick models and colored in red and blue, respectively. Selected side chains involved in Syt1 binding mentioned in the text are labeled. N indicates the N‐terminus of syntaxin‐1 and C the C‐termini of the SNARE motifs of syntaxin‐1, SNAP‐25 and synaptobrevin.

SNARE proteins are characterized by ~ 65‐residue sequences that are known as SNARE motifs and have high propensity to form coiled coils [62]. Synaptobrevin and syntaxin‐1 each contain one SNARE motif preceding a transmembrane (TM) region (Fig. 2A) and are anchored on the vesicle and plasma membranes, respectively, whereas SNAP‐25 has two SNARE motifs connected by a long linker that is palmitoylated at cysteine residues, which mediates anchoring on the plasma membrane [63]. The four SNARE motifs mediate formation of the SNARE complex, which consists of a bundle of four parallel helices rich in charged residues (Fig. 2B). The SNARE complex is highly stable [64] and assembly of the complex brings the two membranes closely together because of the proximity of the synaptobrevin and syntaxin‐1 SNARE motifs to their respective TM regions. These observations led to the proposal that SNARE complexes constitute the engines for membrane fusion [11], which was supported by reconstitution experiments with SNARE‐containing liposomes [65]. Moreover, most types of intracellular membrane traffic depend on SNARE proteins homologous to the neuronal SNAREs [63]. These and many other findings led to the generally accepted notion that most types of intracellular membrane fusion are mediated by analogous SNARE complexes [2, 3, 63]. At synapses, neuronal SNARE complexes are believed to be assembled in steps that start at the N‐terminus (see below), generating a primed state where SNARE complexes are partially assembled to be ready for fast fusion, which occurs when final ‘zippering’ of the SNARE four‐helix bundle at the C‐terminus is triggered upon Ca^2+^ influx [34].

When interpreting biochemical data on SNAREs, it is important to consider that SNARE motifs tend to be promiscuous and can form non‐cognate SNARE complexes. The syntaxin‐1 SNARE motif is a particularly good example, as shown by the many interactions with other proteins (more than 40) that have been reported for this motif (reviewed in Ref. [63, 66]), most of which are unlikely to have physiological relevance. This promiscuity arises in part because the abundance of hydrophobic and charged residues in the syntaxin‐1 SNARE favor nonspecific interactions, and in part because the syntaxin‐1 SNARE motif has a propensity to be incorporated into non‐cogante four‐helix bundles. The latter tendency is illustrated by the observation that syntaxin‐1 and SNAP‐25 form complexes that consist of a four‐helix bundle and have a 2 : 1 stoichiometry because a second copy of the syntaxin‐1 SNARE motif replaces synaptobrevin [67]. Syntaxin‐1‐SNAP‐25 complexes, often called t‐SNARE complexes, were originally thought to serve as receptors for synaptobrevin to form SNARE complexes. However, as described below in the section on ‘Munc18‐1‐SNARE interactions’, it now seems clear that SNARE complex assembly in neurons proceeds through the Munc18‐1‐syntaxin‐1‐synaptobrevin template complex. Important functions in this pathway are played by a long N‐terminal region that is present in syntaxin‐1 and includes a short N‐terminal sequence called the N‐peptide [68, 69], a three‐helix bundle called the H_abc_ domain [48] and a linker region connecting the H_abc_ domain to the SNARE motif (Fig. 2A). A key property of this N‐terminal region is that it forms a closed conformation where the SNARE motif folds back onto the H_abc_ domain, thus hindering SNARE complex assembly [19]. The functional importance of this property was demonstrated by the observation that a L165A,E166A mutation (called LE mutation) that opens syntaxin‐1 [19] enhances the rate of neurotransmitter release [70] and partially rescues the defects in neurotransmitter release caused by deletion of a variety of proteins involved in synaptic transmission [18, 71, 72].

## Interactions of complexins with SNAREs and membranes

Complexins are small soluble proteins that bind tightly to the SNARE complex [42] and play dual roles in release. Thus, in the absence of complexins, Ca^2+^‐evoked neurotransmitter release is impaired in vertebrates and invertebrates, whereas spontaneous release is strongly enhanced in invertebrates and altered to distinct extents in different preparations of vertebrate neurons, ranging from mild decrease to considerable enhancement [73, 74, 75, 76, 77, 78, 79]. This variability in the changes in spontaneous release likely arise from different balances between inhibitory and stimulatory interactions of complexins [80].

**Fig. 3:**
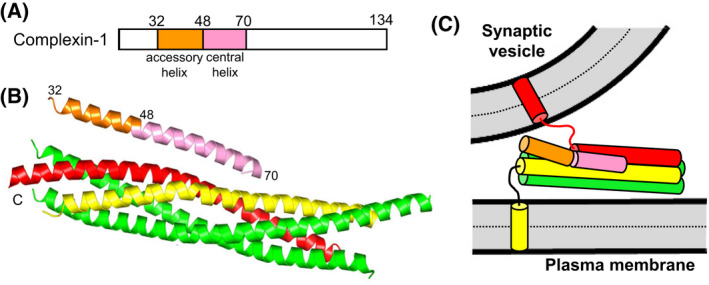
Complexin‐1 structure and function. (A) Domain diagram of complexin‐1. Selected residue numbers are indicated above the diagram. (B) Ribbon diagram of the crystal structure of Cpx1(26–83) (accessory helix in orange, central helix in pink) bound to the SNARE complex, with synaptobrevin in red, syntaxin‐1 in yellow and SNAP‐25 in green [43] (PDB accession number 1KIL). Selected residue numbers are indicated. C denotes the C‐termini of the SNAREs. (C) Model showing how the accessory helix of complexin‐1 bound to a partially assembled trans‐SNARE complex is expected to have steric clashes with the vesicle that would hinder final C‐terminal zippering of the SNARE four‐helix bundle [84].

Complexin‐1, one of the two most abundant complexin isoforms in vertebrates, forms a long α‐helix upon binding to the SNARE complex that is oriented in an antiparallel fashion with respect to the SNARE helices and binds at the groove between the synaptobrevin and syntaxin‐1 SNARE motifs [43] (Fig. 3A,B). In solution, complexin‐1 is largely unstructured, but substantial helical conformation is adopted by the sequence corresponding to the accessory helix [81], suggesting that this sequence helps nucleate helical conformation in the sequence corresponding to the central helix. All complexin functions are abolished by mutations in the central helix that abrogate SNARE complex binding [74, 77], showing the essential functional importance of SNARE complex binding through the central helix.

Mutagenesis also showed that the accessory helix is key for the inhibitory activity of complexins [74, 77, 78, 79, 80, 82]. This inhibition was proposed to arise because, in primed vesicles with partially assembled SNARE complexes, the accessory helix of complexin might replace synaptobrevin at the C‐terminus of the SNARE four‐helix bundle, thus hindering final zippering of the SNARE complex [74]. A related popular model emerged from a crystal structure of a truncated SNARE complex lacking the C‐terminus of the synaptobrevin SNARE motif bound to a complexin‐1 mutant fragment in which three charged residues of the accessory helix were replaced with hydrophobic residues (referred to as superclamp mutant) [83]. The existence of this interaction in solution for both wild type (WT) complexin‐1 and the superclamp mutant was supported by ITC data [83]. However, no interaction of the complexin‐1 accessory helix with C‐terminally truncated SNARE complexes was observed by solution NMR spectroscopy or by ITC in subsequent studies [84]. Moreover, the superclamp mutation was found to have only slightly stimulatory or no effects on neurotransmitter release in mice instead of the enhanced inhibition of release predicted by the model [84]. The relatively small heat observed by ITC in the original experiments was later found to arise from nonspecific interactions involving a His_6_‐tag and the polybasic juxtamembrane sequence following the syntaxin‐1 SNARE motif, which were not present in the crystal structure [85]. These results emphasized that, whereas ITC provides a very powerful tool to quantitatively analyze the thermodynamics of strong protein–protein interactions, the observation of small heats by ITC can arise from unexpected weak interactions involving impurities or promiscuous sequences prone to nonspecific interactions. Hence, alternative tools should ideally be used to verify that weak protein–protein interactions observed in crystal structures can be observed in solution and do not simply constitute irrelevant crystal contacts.

Another model that explains the inhibitory activity of the complexin accessory helix postulates that binding of the central helix to the SNARE complex orients the accessory helix toward the vesicle membrane, causing steric clashes that hinder C‐terminal zippering of the SNARE four‐helix bundle (Fig. 3C). This model was supported by electrophysiological experiments in mouse neurons [84] and by the observation that replacement of the complexin accessory helix with an unrelated sequence with high helix propensity preserves the inhibitory function of complexin in *Caenorhabditis elegans* [82], as this result strongly suggests that the inhibitory activity does not arise from specific protein interactions. Conversely, some evidence suggested that weak interactions of the complexin accessory helix with SNAP‐25 and synaptobrevin inhibit release [86]. It is plausible that transient complexin‐SNARE interactions that do not necessarily need to be specific may hinder C‐terminal zippering of the four‐helix bundle, and that such interactions may occur regardless of the specific sequence of the accessory helix. Molecular dynamics (MD) simulations have provided some support for this view and particularly for the steric hindrance model [87], as discussed below in the section on ‘The complexity of studying synaptotagmin‐1‐SNARE interactions and their coupling with complexins’. Irrespective of the underlying mechanism, strong experimental evidence has supported the notion that complexin hinders C‐terminal zippering of trans‐SNARE complexes [88, 89], even though in solution complexin‐1 binding stabilizes the C‐terminus of the SNARE complex [43].

Complexin‐1 also contains N‐ and C‐terminal regions that are unstructured in isolation [81] but play important functions. The N‐terminal region was found to be critical to relieve the inhibitory activity of the accessory helix in mice [74, 90], but appears to be dispensable in *C. elegans* [78]. This region can form an amphipathic α‐helix and exhibits weak interactions with the N‐terminus of the SNARE complex [90] and with membranes that may cooperate with each other [91]. These interactions are important for Ca^2+^‐induced activation of liposome fusion in reconstitution experiments [91], but the technical difficulties of characterizing their structural basis have hindered elucidation of their mechanism of action. The C‐terminal region of complexin binds to lipids [92] through two motifs that can also form amphipathic α‐helices [93]. It is noteworthy that no binding of complexin‐1 to 100–200 nm liposomes was observed by NMR spectroscopy [90]. This finding was later reconciled by the observation that strong membrane curvature favors binding to the complexin C‐terminal region, which is believed to target complexins to synaptic vesicles [94, 95], illustrating how differences in experimental conditions can lead to different conclusions. It is also important to note that, even if interactions of the complexin C‐terminal region with membranes are weak, they can cooperate with binding to the SNAREs and thus increase the overall affinity, as shown in studies of the Syt1‐complexin interplay described below.

It is still unclear how the various interactions of complexin are integrated and what is the basis for the stimulatory function of complexin in neurotransmitter release. The finding that complexin‐1 strongly protects trans‐SNARE complexes from disassembly by NSF/αSNAP [16] suggests that complexins may protect against de‐priming or at least may help to maintain a larger number of trans‐SNARE complexes assembled such that the release probability is increased. This notion is supported by the finding that, although the absence of complexins does not alter the readily‐releasable pool (RRP) of vesicles, it does increase the sensitivity of release to hypertonic sucrose concentrations below the 500 mm concentration that is typically used to measure the RRP [90].

## Synaptotagmin‐1‐membrane interactions

**Fig. 4:**
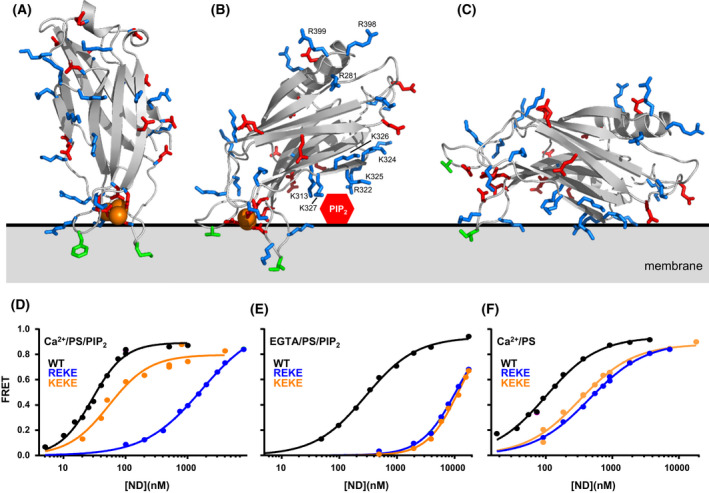
Structure and membrane interactions of the Synaptotagmin‐1 C_2_ domains. (A, B) Ribbon diagrams of the NMR structures of the Syt1 C_2_A (A) and C_2_B (B) domains [47, 51] bound to Ca^2+^ (orange spheres) (PDB accession numbers 1BYN and 1K5W, respectively) in the approximate orientations with respect to a flat phospholipid bilayer (gray) defined by EPR [105, 107]. The slightly slanted orientation of the C_2_B domain in (B) enables interactions of residues from the polybasic region with PIP_2_ head groups that protrude from the bilayer surface (red hexagon). Basic residues involved in SNARE complex and membrane interactions are labeled and shown by stick models. Hydrophobic side chains that insert into the membrane are shown as green stick models. Note that K313, R322, K325 and K327 in the polybasic face of the C_2_B domain can readily bind to PIP_2_ in these orientations whereas K324 and K326 are oriented away from the membrane, farther from the PIP_2_ head group. These observations explain the selective disruption of neurotransmitter release caused by mutations in the polybasic face [108, 112]. (C) Approximately parallel orientation of the C_2_B domain with respect to the membrane expected in the absence of Ca^2+^, which is supported by MD simulations [87]. (D–F) Selectively strong disruption of Syt1 C_2_AB binding to membranes by the R322E,K325E (REKE) mutation but not by the K324E,K326E (KEKE) mutation is observed in the presence of Ca^2+^ and PIP_2_ but not in the absence of Ca^2+^ and/or PIP_2_. The plots show binding of C_2_AB to nanodiscs containing PS and PIP_2_ (D, E) or PS but not PIP_2_ (F) in the presence of Ca^2+^ and 125 mm KCL (D, F) or EGTA and 50 mm KCl (E). These data were reported in [45]. The data in panel (E) were acquired at lower ionic strength because binding is very weak in the absence of Ca^2+^.

Syt1 is a synaptic vesicle protein with two C_2_ domains (named C_2_A and C_2_B) that form most if its cytoplasmic region. Both domains form β‐sandwich structures that bind three (C_2_A) or two (C_2_B) Ca^2+^ ions through five aspartate residues located in loops at one tip of the β‐sandwich structures and contain abundant charged residues [46, 47, 51, 96, 97] (Fig. 4A,B). In particular, the C_2_B domain contains many basic residues, including basic clusters in a polybasic region on the side of the β‐sandwich and an Arg‐rich region at the tip of the β‐sandwich opposite to the Ca^2+^‐binding loops (Fig. 4B). Because of these properties, the C_2_B domain is highly promiscuous and has a high tendency to bind polyacidic contaminants that are not detected by SDS/PAGE and need to be removed through careful purification, including ion exchange chromatography [98, 99]. Results obtained with improperly purified C_2_B domain have muddled the Syt1 literature and readers should consider how Syt1 fragments containing the C_2_B domain were purified when examining published data. For instance, while the Syt1 C_2_A domain was shown early to bind to negatively charged phospholipids in a Ca^2+^‐dependent manner [100], this property was masked in initial studies of the Syt1 C_2_B domain, and Ca^2+^‐dependent phospholipid binding was observed only when the C_2_B domain was properly purified [51]. Phospholipid binding is induced by an electrostatic switch in the Ca^2+^‐binding region of the C_2_ domains, which has a high density of negative charge in the absence of Ca^2+^ and becomes strongly positive after Ca^2+^ binding [51, 101], in part because of basic residues that decorate the Ca^2+^‐binding loops (Fig. 4A,B). Binding is stabilized not only by interactions of these basic residues with the phospholipids but also by insertion of hydrophobic side chains into the acyl region of the membrane bilayer and by partial coordination of Ca^2+^ ions bound to the C_2_ domains by the phosphate groups of the lipids [102, 103]. These findings allowed the design of mutations that decreased or enhanced the apparent Ca^2+^ affinity of Syt1 in phospholipid binding and led to parallel changes in the Ca^2+^ sensitivity of neurotransmitter release, which demonstrated that Syt1 is the major Ca^2+^ sensor that triggers release [37, 38].

The Syt1 C_2_B domain also binds to membranes containing phosphatidylinositol 4,5‐bisphosphate (PIP_2_) in a Ca^2+^‐independent manner, which is mediated by the polybasic region on the side of the β‐sandwich and is believed to steer Syt1 to the plasma membrane [104]. This interaction is expected to occur with the C_2_B domain oriented in an approximately parallel fashion with respect to the plane of the membrane (Fig. 4C), which contrasts with the roughly perpendicular orientation adopted by the C_2_A domain upon Ca^2+^‐dependent membrane binding defined by electron paramagnetic resonance (EPR) [105] (Fig. 4A). The Ca^2+^‐bound C_2_B domain binds to membranes lacking PIP_2_ in a similar perpendicular orientation [106], but the orientation is more slanted when the Ca^2+^‐saturated C_2_B domain binds to PIP_2_ containing membranes such as the plasma membrane [107] to enable interactions of the polybasic region with PIP_2_ head groups, which protrude from the membrane bilayer [45] (Fig. 4B). The apparent Ca^2+^ affinity of Syt1 in membrane binding is enhanced by the presence of PIP_2_ in the membrane [108, 109, 110] and, in turn, PIP_2_ enhances the affinity of Ca^2+^‐bound Syt1 for membranes [45]. There is also synergy between PIP_2_ and negatively charged phospholipids such as phosphatidyl serine (PS) which enhances the membrane affinity for Ca^2+^‐bound Syt1 [111], resulting in a very high affinity. For instance, the *K*_d_ of a fragment spanning both Syt1 C_2_ domains (C_2_AB) for PIP_2_‐PS‐containing nanodiscs in the presence of Ca^2+^ is ~ 20 nm [45] (Fig. 4D). Importantly, a K326A, K327A mutation in the polybasic region that decreased the apparent Ca^2+^ affinity of Syt1 for PIP_2_‐containing liposomes led to a parallel decrease in the Ca^2+^ sensitivity of neurotransmitter release [108]. Moreover, a R322E,K325E mutation (REKE) in the polybasic region disrupts Ca^2+^‐dependent binding to PIP_2_‐containing nanodiscs much more severely than a K324E,K326E mutation (KEKE) [45] (Fig. 4D), which correlates with the strong disruption of neurotransmitter release caused by the R322E,K325E mutation and the mild effects of the K324E,K326E mutation on release [112]. These observations can be readily explained by the fact that, in the orientation of Ca^2+^‐saturated C_2_B domain on PIP_2_‐containing membranes, the PIP_2_ headgroups can readily interact with some of the residues of the polybasic region (e.g., R322, K325 and K327) but not with others (e.g., K324 and K326) (Fig. 4B). It is important to note that this correlation does not apply to Ca^2+^‐independent binding of C_2_AB to PIP_2_‐PS‐containing nanodiscs or Ca^2+^‐dependent binding of C_2_AB to PS‐containing nanodiscs (i.e., lacking PIP_2_), which are similarly disrupted by the R322E,K325E and K324E,K326E mutations [45] (Fig. 4E,F).

These results do not rule out the possibility that Ca^2+^‐independent interactions of Syt1 with PIP_2_ are physiologically relevant but strongly suggest that Ca^2+^‐dependent interactions of selected residues from the C_2_B domain polybasic region with the plasma membrane involving PIP_2_ play a critical role in Ca^2+^‐triggering of neurotransmitter release. Moreover, these findings illustrate how experimental conditions and the presence or absence of key components can have a dramatic effect on the affinities of interactions and the relative impact of mutations on the affinities, emphasizing the importance of systematic analyses to understand the contributions of the different components to binding.

The Arg‐rich region at one tip of the C_2_B domain (Fig. 4B) can also interact weakly with membranes [45] and, when such interaction occurs simultaneously with Ca^2+^‐dependent binding of the loops at the other tip of the C_2_B domain to another membrane, the two membranes are brought into close apposition [113, 114]. Since this activity is Ca^2+^ dependent, these findings led to an attractive model whereby Syt1 cooperates with the SNAREs to bring the synaptic vesicle and plasma membranes together to trigger neurotransmitter release [113]. This model was supported by the observations that an R398Q,R399Q mutation in the Arg‐rich region impairs the ability of the Syt1 C_2_B domain and C_2_AB fragment to cluster liposomes through this membrane‐bridging activity and strongly disrupts neurotransmitter release [115]. Subsequent results provided further support for this model [116]. However, as discussed below, the physiological relevance of these findings remains unclear, particularly after the discovery that R398 and R399 are important for binding to the SNARE complex, and the SNAREs were also found to bind to the Syt1 C_2_A domain and the C_2_B domain polybasic region. Hence, the studies of Syt1‐SNARE interactions described in the next section were critical to sort out which are the physiological targets of the various functional regions of the Syt1 C_2_ domains.

## The complexity of studying synaptotagmin‐1‐SNARE interactions and their coupling with complexins

Studies of Syt1‐SNARE interactions started 30 years ago and research on these interactions is still ongoing, providing a particularly vivid illustration of the difficulties of studying the mechanism of neurotransmitter release. Syntaxin‐1 was originally isolated in screens for synaptic vesicle proteins that bind to Syt1 [117], although it is still unclear whether this result arose from physiologically relevant interactions or from the promiscuity and abundance of both proteins. Subsequent early studies with recombinant proteins reported interactions of the Syt1 C_2_A domain, the C_2_B domain or the C_2_AB fragment with syntaxin‐1, SNAP‐25, syntaxin‐1‐SNAP‐25 heterodimers or the SNARE complex, and most of these interactions either required Ca^2+^ or were strongly enhanced by Ca^2+^, but Ca^2+^‐independent binding was observed in some cases [101, 118, 119, 120, 121, 122, 123]. In these studies, the Ca^2+^ dependence of the interactions was normally associated with the C_2_A domain, whereas the C_2_B domain was responsible for Ca^2+^‐independent binding. NMR studies showed that the Ca^2+^‐binding loops of the C_2_A domain bind in a Ca^2+^‐dependent manner to an acidic region of the syntaxin‐1 H_abc_ domain [48, 101], and the C_2_A domain was also found to bind in a Ca^2+^‐dependent manner to the syntaxin‐1 SNARE motif [121, 124]. Since Ca^2+^ binding to Syt1 is expected to occur in the last step of release, when SNARE complexes are at least partially assembled, the key question that arose was whether Ca^2+^‐dependent binding to the SNARE complex could occur simultaneously with Ca^2+^‐dependent membrane binding. Using a 1D NMR assay developed to analyze protein interactions at low micromolar concentrations, liposomes were shown to compete with the soluble SNARE complex for binding to Syt1 C_2_AB in the presence of Ca^2+^, displacing the SNARE complex [125]. These results suggested that the Ca^2+^‐dependent interactions of Syt1 with the SNAREs are not specific and arise because the SNAREs have abundant acidic regions (Fig. 2B) and the Ca^2+^‐binding regions of the Syt1 C_2_ domains become highly positively charged upon Ca^2+^ binding. Since these regions also bind to the lipids, it was natural that SNARE binding and lipid binding were incompatible.

Nevertheless, some evidence indicated that Syt1 C_2_AB could bind simultaneously to the SNARE complex and lipids [121], and it was plausible that weak Syt1‐SNARE complex interactions not involving the Ca^2+^‐binding regions of the C_2_ domains might be strengthened by co‐localization if the SNARE complex is anchored on a membrane and Ca^2+^ induces binding of the Syt1 C_2_ domains to the same membrane. This hypothesis was tested using partition assays based on microfluidics and confocal fluorescence microscopy [126]. For these assays, supported lipid bilayers containing or lacking anchored SNARE complexes were deposited in separate microchannels and fluorescently labeled Syt1 C_2_AB was allowed to partition between them. Under the conditions of the experiments, Syt1 C_2_AB did not bind to either of the supported bilayers in the absence of Ca^2+^, but partitioned quantitatively to the supported bilayer containing SNARE complexes in the presence of Ca^2+^, demonstrating that Ca^2+^‐saturated C_2_AB indeed bound simultaneously to the SNARE complex and the lipids [126]. A related approach showed that the Syt1 C_2_AB displaced a complexin‐1 fragment spanning the accessory and central helices [Cpx1(26–83)] from membrane‐anchored SNARE complexes [39] and that this activity required the polybasic region of the C_2_B domain as well as two residues from an acidic patch of the C‐terminus of SNAP‐25 (D186 and D193, Fig. 2B) [126] that had been previously implicated in Ca^2+^‐dependent Syt1‐SNAP‐25 interactions [127]. Note also that Syt1 C_2_AB could not displace full‐length complexin‐1 from membrane‐anchored SNARE complexes, most likely because interactions of the complexin‐1 N‐ and C‐terminal regions with membranes cooperate with SNARE complex binding and strengthen the overall affinity [128]. Overall, these and other results supported a popular model whereby Ca^2+^‐dependent binding of Syt1 to the SNARE complex relieves the inhibitory activity of complexins ([39, 126]; see also [40, 41] for related models), which might not require full displacement of complexin‐1 but just a conformational rearrangement of SNARE complex bound complexin‐1 caused by Syt1 binding and steric clashes of complexin‐1 with the membrane [112, 128].

To make matters even more confusing, gel filtration experiments showed that Cpx1(26–83) or full‐length complexin‐1 can bind simultaneously with C_2_AB to soluble SNARE complex [128], even though competition between full‐length complexin‐1 and C_2_AB for SNARE complex binding was observed in pull‐down assays [39]. Both sets of experiments were performed in the presence of Ca^2+^. Multiple observations clarified this apparent paradox. First, the SNARE complex has a high tendency to aggregate, which is exacerbated in the presence of C_2_AB and Ca^2+^ through nonspecific interactions that likely dominated the binding observed in pull‐down assays and were hindered by complexin‐1 because it hinders aggregation of the SNARE complex [128]. Second, the gel filtration experiments were performed with SNARE complex bearing a short C‐terminal truncation in syntaxin‐1 that hinders SNARE complex aggregation [43] and the formation of C_2_AB‐SNARE complex aggregates [128]. Third, there are at least two binding sites for the C_2_AB on the SNARE complex that are distal from the C‐terminus and are compatible with complexin‐1 binding to SNARE complex (see below), which explains the co‐elution of C_2_AB, complexin‐1 and the slightly truncated SNARE complex in gel filtration. And fourth, the proximity of the SNAP‐25 acidic sequence containing D186 and D193 to the C‐terminus (Fig. 2B), which is close to the membrane for membrane‐anchored SNARE complex, likely favors binding of this sequence to membrane‐bound C_2_AB over other binding modes in the competition assays between C_2_AB and Cpx1(26–83) performed on supported bilayers [126]. Indeed, displacement of Cpx1(26–83) by C_2_AB from membrane‐anchored SNARE complex did not involve another acidic sequence of SNAP‐25 including D51, E52 and E55, which is further from the C‐terminus [126] (Fig. 2B) and is involved in the two binding modes defined structurally with soluble proteins (see below). Multiple studies implicated the polybasic sequence in binding to soluble SNARE complexes or syntaxin‐1‐heterodimers [129, 130, 131], but some evidence showed that there was at least one additional SNARE complex binding site in the C_2_B domain [126, 131] and that R398, R399 participated in such binding [99].

**Fig. 5:**
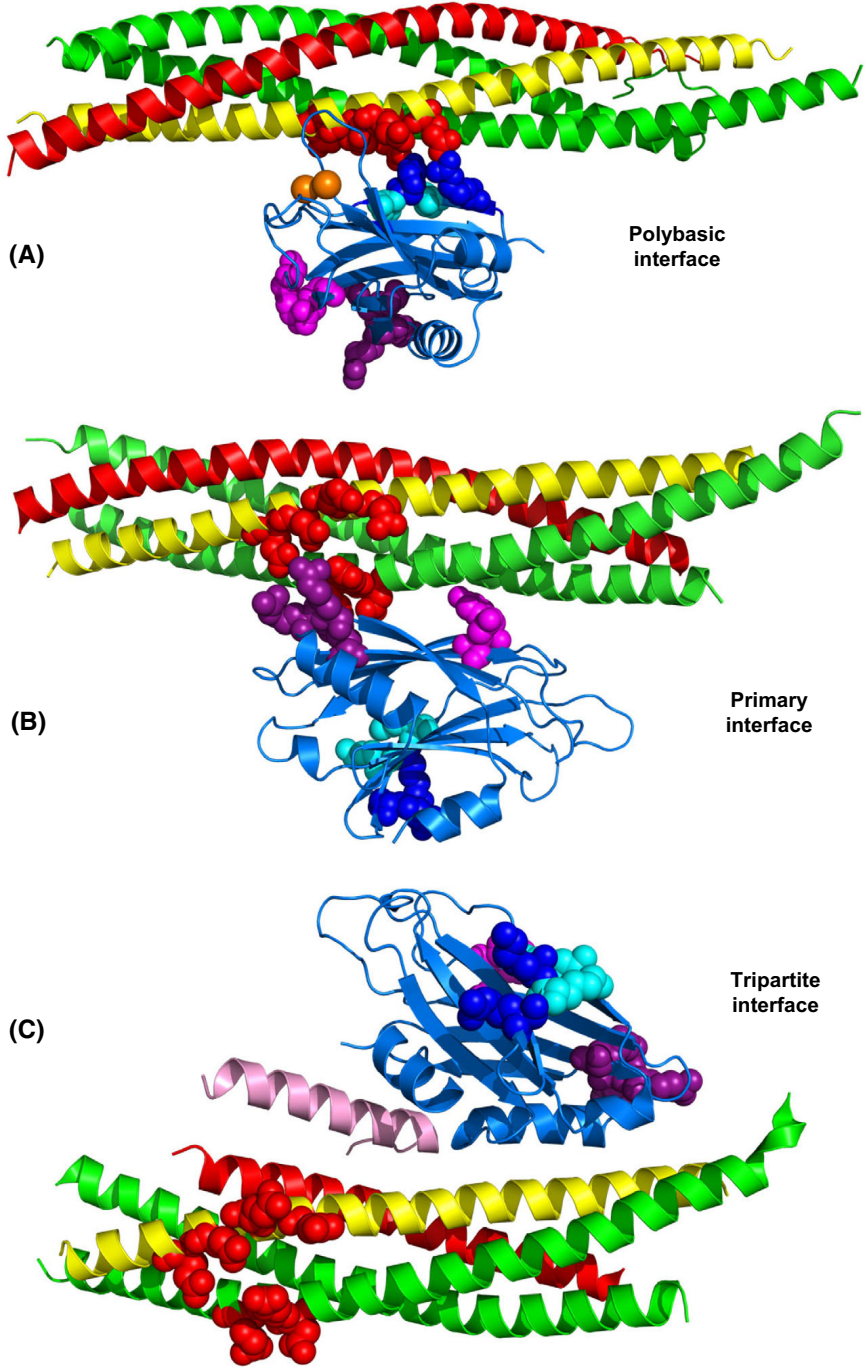
Structures of Synaptotagmin‐1‐SNARE complexes. (A–C) Ribbon diagrams of the dynamic structure of a C_2_B domain‐SNARE complex determined in solution by NMR spectroscopy [112] (A) (only a representative conformer of the large ensemble is shown), of a C_2_B domain‐SNARE complex determined by X‐ray crystallography [44] (B), and of a C_2_B domain‐SNARE‐complexin‐1 complex determined also by X‐ray crystallography [138] (C). The PDB accession numbers are 2N1T, 5KJ7 and 5W5C, respectively. The Syt1 C_2_B domain is in blue, complexin‐1 in pink, synaptobrevin in red, syntaxin‐1 in yellow and SNAP‐25 in green. Selected residues from the C_2_B polybasic region are shown as blue (R322 and K325) or cyan (K324 and K326) spheres, selected C_2_B residues that form the primary are shown as pink (E295 and Y338) or deep purple (R281, R398 and R399) spheres, and selected acidic residues of the SNAREs involved in binding to the polybasic region or the primary interface of the C_2_B domain (D51, E52 and E55 of SNAP‐25, and E224, E228, D231 and E234 of syntaxin‐1) are shown as red spheres.

Characterizing the structural basis of the underlying binding modes was hindered not only by binding heterogeneity but also because the combination of binding modes led to a strong tendency of Syt1‐SNARE complexes to precipitate in the presence of Ca^2+^ at the high concentrations normally required for structure determination. This problem was addressed in an NMR study using KSCN, a chaotropic agent that impedes nonspecific interactions [112]. Measurement of lanthanide‐induced pseudocontact shifts in the presence of KSCN and Ca^2+^ allowed elucidation of a highly dynamic Syt1 C_2_B domain‐SNARE complex structure that was compatible with complexin‐1 binding to the SNAREs. In the broad ensemble of this dynamic structure, the polybasic region of the C_2_B domain binds to the acidic sequence including D51, E52 and E55 of SNAP‐25 in the middle of the SNARE four‐helix bundle or a nearby acidic sequence of syntaxin‐1 including E224, E228, D231 and E234 (Fig. 5A; see also acidic regions in Fig. 2B). The strong disruption of C_2_AB binding to the SNARE complex caused by K313E,K325E and R322E,K325E mutations in the polybasic region, and the milder effects caused by the K324E,K326E mutation (also in the polybasic region) or a control K354E,R388E mutation, were consistent with this dynamic structure and correlated with the effects of these four mutations on neurotransmitter release, whereas these effects did not correlate with those induced by the mutations on Ca^2+^‐independent binding of C_2_AB to PIP_2_‐containing liposomes [112].

These observations strongly supported the physiological relevance of the dynamic C_2_B‐SNARE binding mode defined by NMR spectroscopy in solution. However, as explained above, subsequent systematic studies of Syt1‐SNARE and Syt1‐lipid interactions using nanodiscs showed that the electrophysiological data could also be explained by the effects of the mutations on Ca^2+^‐dependent binding of Syt1 to PIP_2_‐containing membranes, which is much tighter than SNARE complex binding [45]. These and other findings described in the next section strongly suggest that the C_2_B‐SNARE complex binding mode defined by the NMR studies is not physiologically relevant and arose because of the absence of the native target of the polybasic region (PIP_2_‐containing membranes) in the experiments, providing a dramatic illustration that results may need to be re‐interpreted even after a clear correlation between biochemical and physiological data was established with four mutations.

## Structural basis for binding of synaptotagmin‐1 to the SNARE complex

A major breakthrough to understand Syt1‐SNARE coupling came from an ingenious approach where Syt1 C_2_AB was covalently linked to different SNAREs using flexible sequences of different lengths and one such chimeric construct led to a crystal structure revealing a binding mode between the Syt1 C_2_B domain and the SNARE complex [44] that was different from that determined by NMR spectroscopy [112]. In the crystal structure, binding was also mediated by the acidic sequence of SNAP‐25 containing D51, E52 and E55, as well as the neighboring acidic sequence of syntaxin‐1, but involved the face of the C_2_B domain β‐sandwich opposite to the polybasic region (Fig. 5B). This interface, which is referred to as the primary interface, was observed in the absence and presence of Ca^2+^ and involved two regions: region I where E295 and Y338 from the C_2_B domain establish polar and hydrophobic contacts with the SNAREs, and region II where three arginines from the Arg‐rich region of the C_2_B domain (R281, R398 and R399) bind to the acidic sequences of SNAP‐25 and syntaxin‐1. The physiological importance of this interface has been demonstrated by the strong disruption of Ca^2+^‐triggered release caused by the R398Q,R399Q mutation in region II [115] as well as by a double mutation in region I (E295A,Y338W) and a quintuple mutation involving both regions (E295A,Y338W,R281A,R398A,R399A) [44]. Moreover, exocytosis was inhibited by a stapled peptide that specifically inhibits binding of Syt1 (and the closely related Syt2) to the SNARE complex through the primary interface [132]. Intriguingly, KO of Syt1 leads to an enhancement of spontaneous release that can be rescued by WT Syt1 and the E295A,Y338W mutant, but not by the R398Q,R399Q and quintuple Syt1 mutants [44], suggesting that the defects in evoked release caused by the mutations arise from distinct types of alterations.

These results were explained by systematic analyses of Syt1‐SNARE complex interactions in solution by NMR spectroscopy and on nanodiscs containing or lacking anchored SNARE complexes by fluorescence resonance energy transfer (FRET) [45]. The NMR data showed that the R398Q,R399Q mutation in the C_2_B domain disrupts binding to the SNARE complex through the primary interface but the E295A,Y338W mutation actually enhances this interaction, likely because the tryptophan introduced by the mutation establishes more extensive contacts with the SNAREs than the native tyrosine. The FRET assays revealed that binding of Syt1 C_2_AB to nanodisc‐anchored SNARE complexes involved at least two types of interactions mediated by the primary interface or the polybasic region, both in the absence and presence of Ca^2+^, but the results depended on the conditions of the experiments. Importantly, when PIP_2_ was included in the membranes and the buffer contained physiological ionic strength and ATP concentrations, the affinity of C_2_AB for nanodiscs and SNARE‐complex nanodiscs was similar in the presence of Ca^2+^. These findings indicated that C_2_AB does not bind to the SNARE complex under these conditions [45], in agreement with previous results [133]. However, C_2_AB still bound to nanodisc‐anchored SNARE complex in the absence of Ca^2+^ even when ATP was present, also consistent with previous data [134], and binding was enhanced by the E295A,Y338W mutation, showing that this Ca^2+^‐independent interaction was dominated by the primary interface. These results can be rationalized from the realization that, in the absence of Ca^2+^, inclusion of PIP_2_ in the nanodiscs should lead to interactions of PIP_2_ with the C_2_B domain polybasic region, hindering interactions of this region with the SNAREs and thus favoring binding of the C_2_B domain to the SNARE complex through the primary interface. However, this binding mode is incompatible with the very tight, Ca^2+^‐dependent interaction of C_2_AB with a PIP_2_‐containing membrane because the approximately perpendicular orientation of the C_2_B domain with respect to the membrane caused by this interaction [107] (Fig. 4B) would lead to strong steric clashes of the SNARE complex with the membrane [45].

The picture that emerges is that the many lines of evidence that during a period of 25 years showed that Ca^2+^ induces or stimulates Syt1‐SNARE binding in solution or on membranes, including the microchannels experiments mentioned above [39, 126], arose because at least one key physiological factor was missing. The enhancement in Syt1‐SNARE affinity by Ca^2+^ in all those experiments, even when binding was mediated by sequences that do not bind Ca^2+^ such as the polybasic region or the primary interface, was only a natural consequence of the increase in overall positive electrostatic potential of the C_2_B domain induced by Ca^2+^ [51] and the fact that binding involves acidic regions of the SNAREs. Such an enhancement can still occur on membranes lacking PIP_2_ and ATP because the avid polybasic region cannot bind to PIP_2_ and hence binds to any SNARE polyacidic region available, and the Arg‐rich region can still bind to the primary interface and/or participate in nonspecific interactions that are hindered by ATP. Considering the history of this field, it might be premature to completely rule out the relevance of all experiments that showed Ca^2+^‐induced Syt1‐SNARE binding but, given the abundance of PIP_2_ on the presynaptic plasma membrane [135] and of ATP in the cytoplasm, the most parsimonious interpretation of the currently available data is that Ca^2+^ causes dissociation of Syt1 from the SNARE complex at synapses.

**Fig. 6:**
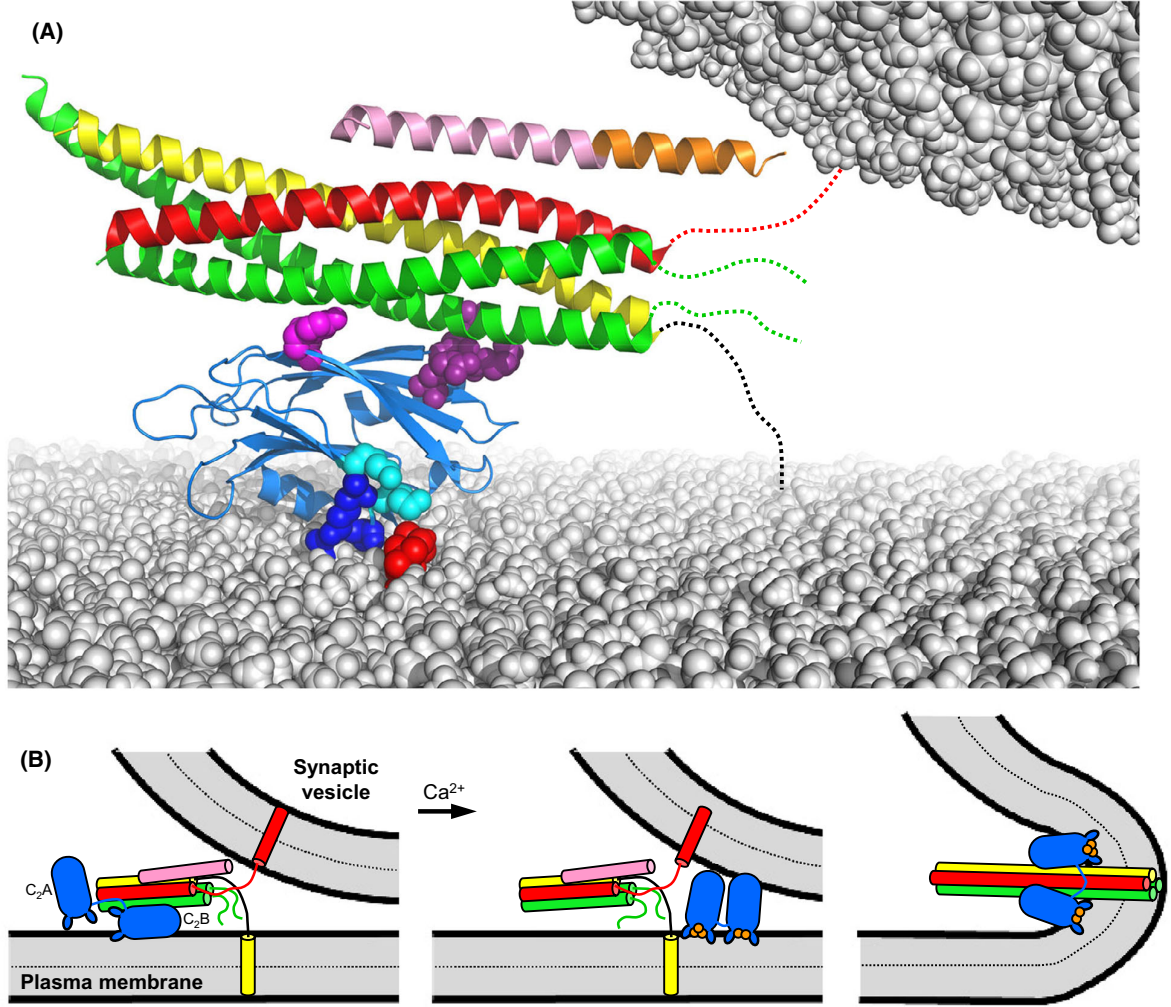
Model of how synaptotagmin‐1, the SNAREs and complexin‐1 form a primed state that prevents premature fusion but is ready for fast membrane fusion upon Ca^2+^ influx. (A) Model of the Syt1‐SNARE‐complexin‐1 primed complex bridging a synaptic vesicle and the plasma membrane before Ca^2+^ influx. The ribbon diagram is based on a superposition of the structures of the Cpx1(26–83)/SNARE complex [43] and of Syt1 C_2_B domain bound to the SNARE complex via the primary interface [44] (PDB accession numbers 1KIL and 5KJ7, respectively). The Syt1 C_2_B domain is in blue, complexin‐1 in orange (accessory helix) and pink (central helix), synaptobrevin in red, syntaxin‐1 in yellow and SNAP‐25 in green. Selected residues from the C_2_B polybasic region are shown as blue (R322 and K325) or cyan (K324 and K326) spheres, and selected C_2_B residues that form the primary are shown as pink (E295 and Y338) or deep purple (R281, R398 and R399) spheres. A PIP_2_ head group in the flat lipid bilayer representing the plasma membrane is shown as red spheres. Dashed lines indicate unstructured regions of the SNARE motif C‐termini that have not zippered because of steric hindrance of the complexin‐1 accessory helix and the vesicle. The flexible linker joining the SNAP‐25 SNARE motifs is not shown for simplicity. (B) Model of Ca^2+^‐triggered neurotransmitter release starting with the primed state present before Ca^2+^ (left), which is analogous to the model of panel (A) but includes the Syt1 C_2_A domain in an arbitrary position. Ca^2+^ influx triggers dissociation of Syt1 from the SNARE complex (middle) and Syt1 and the SNAREs trigger fast membrane fusion (right) by a mechanism that remains highly enigmatic. The complexin‐1 N‐ and C‐terminal regions, and the linker joining the SNAP‐25 SNARE motifs, are not shown for simplicity. This figure is adapted from fig. 10 of Ref. [45].

It is important to note that the interaction of Syt1 with the SNARE complex through the primary interface is relatively weak in solution (*K*_d_ likely larger than 20 μm [45]), but can be enhanced by co‐localization in the restricted volume of this system and by cooperativity with binding of the polybasic region to PIP_2_‐containing membranes in the absence of Ca^2+^, which allows parallel orientations of the C_2_B domain (Fig. 4C) that are compatible with the primary interface (Fig. 6A). In this arrangement, simultaneous binding of complexin‐1 to the opposite side of the SNARE four‐helix bundle orients the accessory helix directly toward the vesicle membrane, which should hinder final C‐terminal zippering of the SNARE complex [84]. This notion implies that Syt1 also contributes to inhibiting release before Ca^2+^ influx because its interactions with the SNAREs and the plasma membrane dictate the orientation of complexin‐1, which has been supported by all‐atom MD simulations [87]. Altogether, these observation led to a model postulating two key features: (a) the complexin‐1‐SNARE‐Syt1 macromolecular assembly constitutes a central feature of the primed state of synaptic vesicles that hinders premature fusion before Ca^2+^ influx but is ready for fast fusion upon Ca^2+^ influx; and (b) Ca^2+^ binding to Syt1 induces a different orientation of the C_2_B domain with respect to the membrane (Fig. 4B), dissociating the inhibitory Syt1‐SNARE interaction and facilitating cooperation between the SNAREs and Syt1 in inducing fast membrane fusion [45] (Fig. 6B). The mechanism by which Syt1 facilitates membrane fusion remains unclear, but it likely arises because Syt1 causes Ca^2+^‐dependent bridging of the vesicle and the plasma membranes [113, 116], induces membrane curvature [113, 136] and/or perturbs the lipid bilayers through insertion of the C_2_ domain Ca^2+^‐binding loops [137].

In this model, the R398Q,R399Q and quintuple mutations in the C_2_B domain disrupt release [44, 115] because Syt1‐SNARE complex binding through the primary interface plays a critical role, perhaps because it helps in vesicle priming, and Syt1 bearing these mutations cannot rescue the inhibitory activity of Syt1 in spontaneous release because it cannot bind to the SNARE complex. Conversely, the E295A,Y338W mutation impairs evoked release [44] because it hinders Ca^2+^‐induced dissociation of Syt1 from the SNAREs, but preserves the ability of Syt1 to inhibit spontaneous release. This model also explains the observation that complexins are required for the dominant negative effect caused by overexpression of Syt1 bearing mutations that abolish Ca^2+^ binding to the C_2_B domain [138]. Moreover, most of the Syt1 mutations that were found to abrogate this dominant negative effect in a screen performed in Drosophila mapped to the primary interface or were expected to cause misfolding [139], supporting the notion that binding through this interface underlies an inhibitory activity that must be released by Ca^2+^ binding to the Syt1 C_2_B domain.

A crystal structure of a Syt1‐SNARE‐complexin‐1 complex revealed yet another Syt1‐SNARE binding mode whereby an α‐helix of the C_2_B domain binds to the groove between the synaptobrevin and syntaxin‐1 SNARE motifs of the SNARE complex, next to the complexin‐1 central helix, which is called the tripartite interface [138] (Fig. 5C). ITC data supported the existence of this interaction in solution and its physiological relevance was supported by the finding that a L387Q,L394Q mutation in the Syt1 C_2_B domain disrupted binding to the SNARE‐complexin‐1 complex, as assessed by ITC, and neurotransmitter release in neurons [138]. However, our laboratory could not detect this interaction by solution NMR spectroscopy [45, 128]. Our laboratory is collaborating with the laboratory of Axel Brunger to clarify this contradictory data. Using Syt1 C_2_B domain purified by our protocol [99], which includes ion exchange chromatography, we have not been able to observe C_2_B binding to complexin‐1‐SNARE complex through the tripartite interface in solution by ITC or by a very sensitive NMR method involving paramagnetic broadening effects (K. Jaczynska, L. Esquivies, R. Pfuetzner, A. Brunger, & J. Rizo, unpublished results). These results again illustrate the limitations of ITC to unambiguously demonstrate weak protein interactions in solution. The available data do not rule out the possibility that binding of Syt1 to the SNARE complex through the tripartite interface is biologically relevant, as it is plausible that this interaction is very weak and is strengthened by cooperativity with other interactions at the synapse. However, such relevance will need to be investigated with further research using methods to analyze protein interactions on membranes and establishing clear correlations between binding and function with multiple mutations.

An intriguing property of Syt1 is the ability to form oligomeric rings that are disrupted by Ca^2+^ binding, which suggested a model whereby formation of these rings inhibits neurotransmitter release [140]. The biological relevance of these rings has been supported by the enhancements of release caused by an F349A mutation that disrupts these rings [141, 142]. However, the F349A mutation did not enhance Ca^2+^‐evoked release in another study [143], and these rings were not observed in cryo‐EM and cryo‐electron tomography (cryo‐ET) images of reconstituted liposomes containing synaptotagmin‐1 [144] (J. Xu & J. Rizo, unpublished results). Note also that F349 is buried in the tripartite interface and the F349A mutation could thus affect this interface. Hence, further research will also be necessary to examine the relevance of these oligomeric rings. An additional aspect that needs to be investigated is the interplay between Syt1 and other Ca^2+^ sensors that may compete with Syt1 for binding to the SNARE complex, such as synaptotagmin‐7 [145] and Doc2b [146]. Clearly, we can expect further twists and turns in the Syt1 saga.

## Munc18‐1‐SNARE interactions

**Fig. 7:**
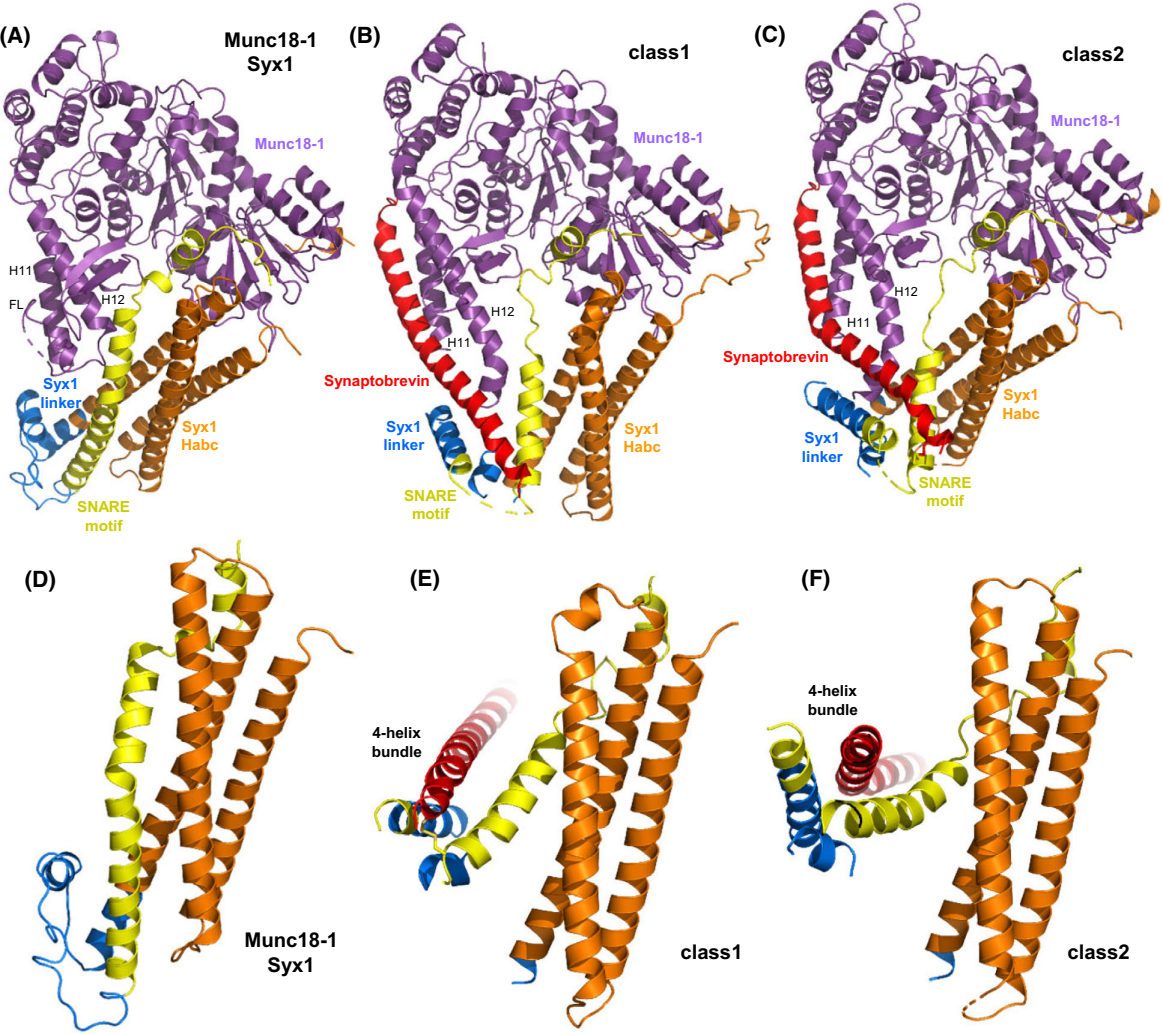
Function of Munc18‐1 in organizing SNARE complex assembly. (A–C) Ribbon diagrams of the crystal structure of the Munc18‐1‐closed syntaxin‐1 complex (A) [20] and the two cryo‐EM structures of the Munc18‐1‐syntaxin‐1‐synaptobrevin template complex (B, C) [33] (PDB accession numbers 3C98, 7UDC and 7UDB, respectively). Syntaxin‐1 is abbreviated Syx1 and the two cryo‐EM structures are denoted class1 and class2. Munc18‐1 is in purple, synaptobrevin in red and syntaxin‐1 in orange (N‐peptide and H_abc_ domain, blue (linker region) and yellow (SNARE motif). The positions of helices 11 and 12 (H11 and H12) of Munc18‐1, and the furled loop that connects these helices and hinders synaptobrevin binding (labeled FL) are indicated. (D–F) Close‐up views of the region where the syntaxin‐1 SNARE motif contacts the H_abc_ domain and the linker in the Munc18‐1‐closed syntaxin‐1 complex (D), class1 (E) and class2 (F). Munc18‐1 is not shown for simplicity. Note how the SNARE motif separates gradually from the H_abc_ domain in the three structures from left to right and how the syntaxin‐1 linker forms a short four‐helix bundle with the syntaxin‐1 and synaptobrevin SNARE motifs in class1 and class2.

Munc18‐1 is a member of a family of soluble proteins that are collectively known as Sec1/Munc18 (SM) proteins and play crucial roles in all forms of intracellular membrane traffic that depend on SNAREs [147]. This crucial nature was particularly well illustrated by the total abrogation of all forms of release observed in Munc18‐1 KO mice, including Ca^2+^‐evoked, spontaneous and sucrose‐induced release (which releases all vesicles from the RRP) [148]. Hence, elucidating the function(s) of Munc18‐1 was critical to understand the mechanism of neurotransmitter release. Achieving this goal was hindered in early studies because of the diversity of interactions between SM proteins and SNAREs that were observed [149]. Munc18‐1 was found to bind tightly to syntaxin‐1 [150] and this tight interaction required the closed conformation of syntaxin‐1 [19]. This conclusion was confirmed by the crystal structure of Munc18‐1 bound to syntaxin‐1, which revealed that Munc18‐1 has an arch‐shaped, three‐domain architecture that wraps around closed syntaxin‐1 [20] (Fig. 7A). Sso1, the yeast homolog of syntaxin‐1, was also shown to adopt a closed conformation [151], but Sec1, the Munc18‐1 homolog involved in exocytosis in yeast, was found to bind to the yeast exocytotic SNARE complex rather than to isolated Sso1 [152]. Moreover, Vam3, the yeast vacuolar syntaxin, did not adopt a closed conformation, and binding of Vam3 to its cognate SM protein Vps33 required the SNARE motif but not the H_abc_ domain [153]. Sed5 and Tlg2, the yeast syntaxins that function at the Golgi and endosomes, bound tightly to their cognate SM proteins Sly1 and Vps45 through the short N‐peptide sequences right at their N‐termini [154, 155]. This divergent picture was later changed to some degree by the observation that neuronal syntaxin‐1 also contains an N‐peptide that binds weakly to Munc18‐1 but enables binding to the SNARE complex in cooperation with other interactions involving the H_abc_ domain and the SNARE four‐helix bundle [68, 69]. The N‐peptide was found to also contribute to the affinity of Munc18‐1 for closed syntaxin‐1 [156].

It now appears that most SM proteins bind to their cognate SNARE complexes through similar modes, whereas the interaction of Munc18‐1 with closed syntaxin‐1 emerged as a specialization for the exquisite regulation of neurotransmitter release [157]. The role of this tight interaction was initially unclear, as Munc18‐1 binding stabilizes the closed conformation and hinders SNARE complex assembly [19, 24, 158], which does not explain why Munc18‐1 is essential for release. However, it later became clear that binding of Munc18‐1 to closed syntaxin‐1 initiates the productive pathway that leads to synaptic vesicle fusion and that also requires Munc13s, which open syntaxin‐1 [7, 24] (see next section). Thus, fusion assays with reconstituted proteoliposomes showed that, although the neuronal SNAREs and synaptotagmin‐1 can induce liposome fusion, as described previously [159, 160], such fusion is abolished by NSF and αSNAP because they disassemble SNARE complexes; however, addition of Munc18‐1 and a Munc13‐1 spanning its conserved C‐terminal region (Munc13C) leads to efficient fusion [7, 21] because they orchestrate assembly of the SNARE complex in an NSF/αSNAP‐resistant manner [16]. Moreover, αSNAP acts as a strong inhibitor of fusion by different mechanisms, and binding of Munc18‐1 to closed syntaxin‐1 is the only way to sequester syntaxin‐1 and bypass the inhibitory activity of αSNAP [15]. Although the syntaxin‐1 closed conformation is not conserved in the yeast vacuolar fusion machinery, the HOPS hexameric complex that includes Vps33 was found previously to mediate SNARE complex assembly in a manner that was resistant to Sec18 and Sec17, the yeast homologs of NSF and SNAPs, respectively [161], suggesting that protection against the SNARE disassembly machinery is a universal activity of SM proteins or SM‐protein containing complexes.

Munc18‐1 was also found to bind weakly to synaptobrevin, but binding appeared to be mediated by the juxtamembrane region linking the SNARE motif and TM region of synaptobrevin, which is highly promiscuous because it contains multiple basic residues and three aromatic residues [28]. Nevertheless, a subsequent study identified a mutation in helix 12 of domain 3a of Munc18‐1 (L348R) that disrupted binding of Munc18‐1 to synaptobrevin as well as the ability of Munc18‐1 to stimulate liposome fusion [29], leading to the proposal that Munc18‐1 forms a template to assemble the SNARE complex. Tantalizing evidence for this proposal came from two crystal structures of yeast vacuolar Vps33 bound to either Vam3 or Nyv1, the yeast vacuolar homolog of synaptobrevin [30]. These structures showed that the C‐terminal halves of the Vam3 and Nyv1 SNARE motifs bind to distal regions of Vps33 but, if both SNAREs were to bind simultaneously in these modes, the N‐termini of their SNARE motifs would be close to each other and in register to initiate SNARE complex assembly. NMR studies later showed that binding of synaptobrevin to Munc18‐1 via the same mode observed in the Vps33/Nyv1 complex was hindered by a loop joining helices 11 and 12 of Munc18‐1, which furls over the two helices and partially occludes the putative synaptobrevin binding site in the closed syntaxin‐1/Munc18‐1 complex [32] (Fig. 7A). Indeed, NMR data showed that a D326K mutation in Munc18‐1 designed to unfurl this loop increased synaptobrevin binding and binding involved most of the SNARE motif, as predicted by homology with the Vps33/Nyv1 complex structure [32]. The same study showed that the D326K mutation led to overt gains of function in liposome fusion assays *in vitro* and in functional assays in *C. elegans*. Moreover, a systematic study using optical tweezers yielded overwhelming evidence that Munc18‐1 acts as a template for SNARE complex assembly through its interactions with synaptobrevin and syntaxin‐1, and, interestingly, showed that the syntaxin‐1 N‐terminal region plays a critical role in the assembly mechanism beyond its participation in forming the closed conformation [31].

Two cryo‐EM structures of a Munc18‐1‐synaptobrevin‐syntaxin‐1 complex recently provided a clear explanation for this observation and for the first time allowed visualization of a template complex with both SNAREs bound simultaneously to an SM protein [33] (Fig. 7B,C). Because the low affinity of synaptobrevin for Munc18‐1 hinders structure determination of their complexes, in this study synaptobrevin was cross‐linked to syntaxin‐1 through a disulfide bond that had been used in the optical tweezer study and is compatible with the structure of the SNARE complex [31]. Although the cross‐link most likely introduced structural bias, this bias was expected to favor productive intermediates in the SNARE complex assembly pathway and, indeed, the cross‐link strongly facilitated SNARE complex formation [33]. Moreover, the functional relevance of the two cryo‐EM structures was confirmed by binding, SNARE complex assembly and liposome fusion assays using a battery of mutants. The structures revealed how the C‐terminal half of the synaptobrevin SNARE motif binds to the groove formed by helices 11 and 12 of Munc18‐1, as observed in the Vps33‐Nyv1 complex, while the C‐terminal half of the syntaxin‐1 SNARE motif binds to a distal pocket of Munc18‐1, as observed in the closed syntaxin‐1‐Munc18‐1 complex (Fig. 7A–C) and in the Vps33‐Vam3 complex. In both cryo‐EM structures, the N‐peptide and H_abc_ domain of syntaxin remain in contact with analogous surfaces of Munc18‐1 as in the Munc18‐1‐syntaxin‐1 complex. Interestingly, the N‐termini of the two SNARE motifs form a small four‐helix bundle with helices formed by the linker between the H_abc_ domain and the SNARE motif, but the extent of contact between the SNARE motif and the H_abc_ domain is different in the two cryo‐EM structures, and substantially lower than in closed syntaxin‐1 bound to Munc18‐1 (Fig. 7D–F). Hence, the three structures illustrate how syntaxin‐1 opens gradually to initiate SNARE complex assembly, which most likely involves binding of SNAP‐25 to the N‐termini of the syntaxin‐1 and synaptobrevin SNARE motifs and release of their interactions with the syntaxin‐1 linker, followed by dissociation of SNARE‐Munc18‐1 interactions.

This mechanism shows how various weak interactions between particular regions of Munc18‐1 and the SNAREs and between sequences of syntaxin‐1 are formed and dissociated at different stages of the SNARE complex assembly pathway. Clearly, multiple energy barriers hinder SNARE complex assembly in this pathway, providing multiple opportunities for regulation of release. Indeed, as discussed in the next section, Munc13‐1 is critical to overcome at least some of these energy barriers and mediates multiple forms of regulation of release. It is also worth noting that the discovery that Munc18‐1 binds to the SNARE complex suggested potential mechanisms by which Munc18‐1 could directly cooperate with the SNAREs in membrane fusion, thus providing an explanation for the essential nature of Munc18‐1 for neurotransmitter release [68]. However, the subsequent results showing the crucial importance of Munc18‐1 in organizing SNARE complex assembly [33], overcoming the inhibitory activity of αSNAP and the SNARE complex disassembly activity of NSF/αSNAP [7, 15, 16], provide compelling explanations for this essential nature and now it seems clear that Munc18‐1 does not associate with the SNARE four‐helix bundle after assembly. Nevertheless, Munc18‐1 likely remains attached to the SNARE machinery through interactions with the syntaxin‐1 N‐peptide and H_abc_ domain, which serve as anchor points to keep Munc18‐1 bound while syntaxin‐1 undergoes the dramatic conformational re‐arrangements that take place for SNARE complex assembly.

## Munc13‐1‐SNARE interactions

The probability of neurotransmitter release is modulated in a variety of short‐ and long‐term presynaptic plasticity processes that shape the properties of neural networks and underlie diverse forms of information processing in the brain [162]. Among the multiple proteins that mediate these processes, Munc13s can be considered as particularly critical master regulators of release because they govern multiple forms of short‐term plasticity and provide a connection to long‐term plasticity processes and other forms of short‐term plasticity through their interactions with Rab‐interacting molecules (RIMs) [163]. In addition, Munc13s play essential roles in release, as shown by the total abrogation of Ca^2+^‐evoked, sucrose evoked and spontaneous release observed in their absence [164, 165, 166, 167], similar to Munc18‐1 KO mice. This essential role and the similarity of the phenotypes observed in the absence of Munc18‐1 and Munc13s was explained, as described above, by reconstitution experiments showing that Munc18‐1 and Munc13‐1 are essential to orchestrate SNARE complex assembly in an NSF/αSNAP‐resistant manner [7, 16].

**Fig. 8:**
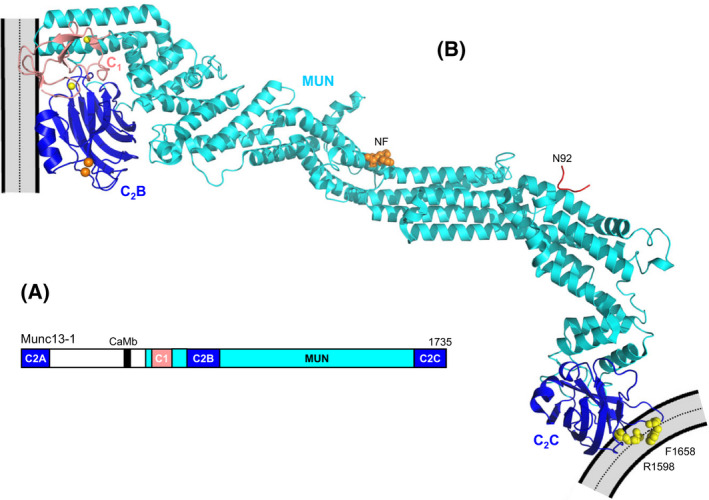
Munc13‐1 bridging a synaptic vesicle and the plasma membrane. (A) Domain diagram of Munc13‐1. The length of Munc13‐1 is indicated by the number on the right, above the diagram. (B) Model of how Munc13‐1 bridges a synaptic vesicle and the plasma membrane in an approximately perpendicular orientation. The ribbon diagram represents one of the structures of Munc13C determined by cryo‐EM of 2D crystals of Munc13C between to phospholipid bilayers [177] (PDB accession code 7T7V) by reconstructing the density map with the help of models from AlphaFold [184] and the crystals structure of Munc13‐1 C_1_C_2_BMUN [176], Ca^2+^‐bound Munc13‐1 C_2_B domain [60] and the MUN domain [25] (PDB accession codes 5UE8, 6NYT and 4Y21, respectively). The C_1_ domain is shown in salmon and the C_2_B and C_2_C domains in blue. Ca^2+^‐ions bound to the C_2_B domain and zinc ions bound to the C_1_ domain are shown orange and yellow spheres, respectively. Residues N1128 and F1131 (NF), which are critical for the activity of the MUN domain in opening syntaxin‐1 [25], are shown as orange spheres, and R1598 and F1658 in the loops of the C_2_C domain, which are crucial for the membrane bridging activity of Munc13C [22], are shown as yellow spheres. A peptide corresponding to the juxtamembrane region of synaptobrevin in the position observed in the crystal structure of this peptide bound to the MUN domain [181] (PDB accession code 6A30) is represented by a red ribbon and its C‐terminal residue (N92) is labeled. Note the large distance from this residue to the vesicle, where the TM region of synaptobrevin, which starts at residue 95, is anchored. The orientation with respect to the flat membrane is approximately that observed in MD simulations in the absence of Ca^2+^ [23].

Munc13‐1 is the most abundant Munc13 isoform in mammalian brain and has a large (200 kDa) multidomain architecture that includes (Fig. 8A): (a) an N‐terminal C_2_A domain that binds to RIMs [168, 169]; (b) a calmodulin‐binding (CaMb) region that mediates some forms of Ca^2+^‐dependent short‐term plasticity [170, 171]; (c) a C_1_ domain involved in diacylglycerol‐ (DAG) and phorbol ester‐dependent augmentation of release [172, 173]; (d) a C_2_B domain that binds Ca^2+^ and PIP_2_ and also mediates short‐term Ca^2+^‐dependent plasticity [60]; (e) a MUN domain involved in opening syntaxin‐1 [24, 25, 174]; and (f) a C_2_C domain that binds weakly to membranes in a Ca^2+^‐independent manner [22]. The N‐terminal region spanning the C_2_A domain and CaMb‐binding region is variable in the Munc13 family, whereas the C‐terminal region spanning the C_1_, C_2_B, MUN and C_2_C domains (Munc13C) is conserved in all Munc13s and is sufficient to rescue neurotransmitter release in Munc13‐1/2 double KO mice [21]. Key steps to understand the function of the C‐terminal region of Munc13‐1 were the identification of the MUN domain [174], which allowed the preparation of various well‐folded fragments spanning parts of the C‐terminal region, and the finding that this domain accelerates the transition from the closed syntaxin‐1/Munc18‐1 complex to the SNARE complex [24]. However, elucidating the underlying mechanism has been hindered because no strong interaction of Munc13‐1 with the SNAREs or Munc18‐1 has been identified.

Analyses by NMR spectroscopy showed that the Munc13‐1 MUN domain binds weakly to Munc18‐1, to the closed syntaxin‐1/Munc18‐1 complex, to the SNARE complex, and to the syntaxin‐1 SNARE motif, and the latter interaction was proposed to be key for the activity of the MUN domain in opening syntaxin‐1 [24]. However, a screen for mutations in syntaxin‐1 that disrupt the ability of the MUN domain to stimulate syntaxin‐1 opening revealed that two residues from the linker between the syntaxin‐1 H_abc_ domain and the SNARE motif (R151 and I155) were critical for such stimulation, and the physiological relevance of this observation was shown by the substantial impairment in synaptic vesicle priming caused by R151A or I155A mutations in syntaxin‐1 [26]. Single molecule FRET assays in this study showed that the MUN domain binds to the closed syntaxin‐1/Munc18‐1 complex and changes the conformation of the linker in the complex. Intriguingly, binding of the MUN domain to the syntaxin‐1 linker region was barely detectable through perturbations in ^1^H‐^15^N HSQC spectra acquired at 20 μm concentration, and evidence for such binding could only be obtained by NMR spectroscopy using paramagnetic broadening effects [27]. These results provide a vehement illustration of the fact that very weak protein interactions can play crucial biological functions, which in this case most likely arises because the MUN domain‐syntaxin‐1 linker interaction decreases the energy barrier to open syntaxin‐1.

Another mutagenesis screen identified two residues of the Munc13‐1 MUN domain (N1128 and F1131) that are also important for stimulation of SNARE complex formation starting from the closed syntaxin‐1/Munc18‐1 complex [25], perhaps by binding to the syntaxin‐1 linker. Interestingly, optical tweezer experiments revealed that the MUN binds to the Munc18‐1‐syntaxin‐1‐synaptobrevin template complex and enhances SNAP‐25 binding [175], suggesting that the MUN domain does more than simply open syntaxin‐1. All these results have provided important insights into how Munc13‐1 cooperates with Munc18‐1 in organizing SNARE complex assembly, but gaining a more detailed understanding of the underlying mechanism is hindered by the lack of a high‐resolution structure of the MUN domain bound to syntaxin‐1.

A crystal structure was determined for an isolated Munc13‐1 fragment spanning its C_1_, C_2_B and MUN domains (C_1_C_2_BMUN), revealing a banana‐shaped architecture in which the C_1_ and C_2_B domains emerge on one end of the highly elongated MUN domain and the C_2_C domain (not present in the fragment) is expected to emerge at the other end [176]. Since the C_1_ and C_2_B domains bind lipids from the plasma membrane (DAG and PIP_2_, respectively) and the C_2_C domain might also bind to membranes, this architecture and liposome clustering assays led to the proposal that the conserved C‐terminal region of Munc13‐1 bridges the synaptic vesicle and plasma membranes [21, 176] (see model of Fig. 8B). Indeed, experiments with a fragment spanning the MUN and C_2_C domains showed that the C_2_C domain binds weakly to membranes, and the Munc13C fragment spanning the C_1_, C_2_B, MUN and C_2_C domains of Munc13‐1 was shown to bridge liposomes by cryo‐ET [22]. Moreover, R1598E and F1658E mutations in the predicted membrane‐binding loops of the C_2_C domain strongly disrupted the liposome bridging activity of Munc13C, the activation of liposome fusion by Munc13C *in vitro* and the ability of Munc13‐1 to rescue neurotransmitter release in Munc13‐1/2 double KO neurons, demonstrating the critical functional importance of the membrane‐membrane bridging activity [22]. The structure of C_1_C_2_BMUN also suggested that Munc13‐1 can bind to the plasma membrane in two orientations: (a) an almost perpendicular orientation resulting from binding through a polybasic face formed by the C_1_ and C_2_B domains, which could allow initiation of SNARE complex formation but would hinder full SNARE complex assembly; and (b) a much more slanted orientation favored by Ca^2+^ binding to the C_2_B domain and DAG binding to the C_1_ domain, which would bring the two membranes into proximity and favor full SNARE complex zippering [176].

Biochemical and electrophysiological experiments have supported this proposal and the notion that tilting the balance between the two orientations underlies the facilitation of neurotransmitter release caused by accumulation of DAG and intracellular Ca^2+^ during short‐term presynaptic plasticity [23]. This study also described MD simulations that yielded models for the two orientations of Munc13‐1 with respect to the membrane. Interestingly, cryo‐EM structures of 2D crystals formed by Munc13C between to lipid bilayers revealed trimers and hexamers with different orientations, one upright and another more slanted [177]. The orientations are not identical to those derived from the MD simulations and there are uncertainties with regard to the physiological relevance of the orientations observed in both studies, as the MD simulations were biased by the initial orientations used and the orientations observed by X‐ray crystallography could be influenced by crystal packing. However, the overall available data strongly supports the notion that at least two distinct orientations of Munc13‐1 bridging the vesicle and plasma membranes exist and can lead to differential effects on neurotransmitter release. The relevance of the trimers and hexamers will also need to be tested through mutagenesis of the corresponding interfaces. Nevertheless, the cryo‐EM structures provide key frameworks to test hypotheses, and the observation of oligomeric structures fits well with imaging studies showing that Munc13‐1 nanoclusters define release sites at synapses [178, 179].

The Munc13‐1 MUN domain was also found to bind to SNAP‐25 [180], but the relevance of this interaction is unclear because binding required the cysteines present in the linker region joining the two SNARE motifs of SNAP‐25, which *in vivo* are palmitoylated to anchor SNAP‐25 on the plasma membrane. Moreover, these cysteines are not required to observe Munc13C‐dependent activation of liposome fusion in reconstitution assays [21]. An additional interaction reported for the MUN domain involved the juxtamembrane region of synaptobrevin, and a crystal structure of the MUN domain bound to a peptide containing the juxtamembrane region led to the proposal that this interaction plays a role in SNARE complex assembly and neurotransmitter release [181]. However, the proximity of the juxtamembrane region to the vesicle membrane is expected to favor interactions of the basic and aromatic residues of this region with the lipids. Moreover, the model of Fig. 8B shows that, based on the crystal structure, residue 92 of synaptobrevin would be very far from the vesicle, a topology that is incompatible with vesicle anchoring of the synaptobrevin TM region, which starts at residue 95. These results provide another illustration of the notion that protein–protein interactions of weak affinity observed in crystal structures need to be interpreted with caution.

## Munc13‐1‐RIM homodimer‐heterodimer switch

**Fig. 9:**
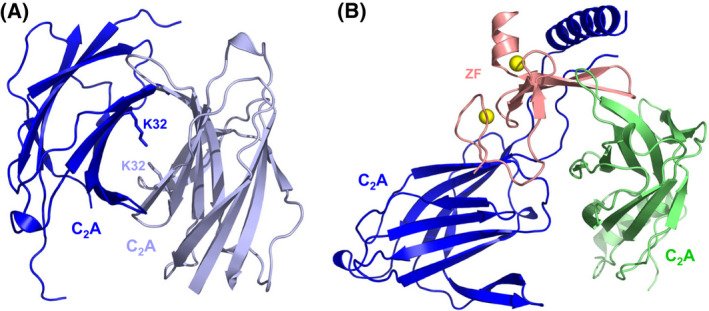
Munc13‐1‐RIM homodimer heterodimer switch. (A, B) Crystal structures of the Munc13‐1 C_2_A domain homodimer (A) and the Munc13‐1 C_2_A domain‐Rim ZF heterodimer (B) [59] (PDB accession numbers 2CJT and 2CJS, respectively). In (A), the K32 side chains in the homodimerization interface are labeled. In (B), the RIM ZF domain is in salmon and the two Munc13‐1 fragments containing the C_2_A domain observed in the crystals are in blue and light green. Zinc ions are shown as yellow spheres. Note that the interface of the blue fragment with RIM is the biologically relevant interface [183], whereas the interfaces formed by the light green fragment arise because of crystal contacts [59].

The Munc13‐1 C_2_A domain forms a homodimer [59], which inhibits neurotransmitter release [182], while binding of an N‐terminal zinc finger (ZF) domain from RIM to the Munc13‐1 C_2_A domain [168, 169] relieves this inhibition and activates release [182, 183]. In addition, the Munc13‐1‐RIM interaction is believed to provide a link between the central machinery that mediates synaptic vesicle fusion and RIM‐dependent forms of presynaptic plasticity [169]. Although the heterodimer has higher affinity than the homodimer, the presence of small amounts of homodimer hindered crystallization of the heterodimer to elucidate the structural basis of the Munc13‐1/RIM interaction [59]. In turn, obtaining high quality diffraction crystals of the Munc13‐1 C_2_A domain homodimer was hindered by its tendency to aggregate. Based on the usefulness of NMR spectroscopy to optimize the SNARE complex for crystallization with Cpx1(26–83) [43], the quality of NMR spectra of different fragments spanning the Munc13‐1 C_2_A domain was used to guide crystallization efforts and eventually led to the crystal structure of the homodimer (Fig. 9A). The structure allowed the design of a mutation (K32E) that disrupted homodimerization and immediately facilitated crystallization of the Munc13‐1 C_2_A domain/RIM ZF heterodimer [59]. Surprisingly, the crystals contained two molecules of the Munc13‐1 fragment containing the C_2_A domain for each RIM ZF molecule (Fig. 9B) even though NMR spectroscopy and ITC unambiguously demonstrated that binding occurred with a 1 : 1 stoichiometry in solution. The C_2_A domain and a C‐terminal helical extension of one of the Munc13‐1 fragments was found to wrap around the RIM ZF (blue ribbon in Fig. 9B) and the biological relevance of this binding mode was supported through mutagenesis and electrophysiology [183]. Thus, the interfaces observed for the second Munc13‐1 fragment (light green in Fig. 9B) clearly constitute irrelevant crystal contacts. It is noteworthy that this 2 : 1 stoichiometry was observed in three different crystal forms and that crystallization was performed with the purified 1 : 1 complex [59]. Hence, the formation of crystal contacts induced the dissociation of half of the tight 1 : 1 heterodimers to yield the 2 : 1 stoichiometry in the crystals. These results provide a dramatic example of the need to interpret protein–protein interactions observed in crystals with caution.

## Perspective

The research summarized above has led to enormous advances in elucidating the molecular mechanisms underlying neurotransmitter release. At the same time, this research has provided and is providing lessons of persistence and innovation from laboratories around the world to investigate a biological process of the utmost interest, integrating approaches from very diverse disciplines. These efforts have led to seminal contributions in many cases and sometimes to studies that needed to be re‐interpreted. However, even the latter studies often moved the field forward because they generated hypotheses that could be tested and/or yielded insights into interactions that needed to be characterized to understand the behaviors of the corresponding proteins, even if the interactions are not biologically relevant. Both the knowledge that has been gained on the mechanisms of action of the release machinery and the technical lessons that have been learned, often more instructive in cases of failure than in success, will be invaluable to make further progress in this field and address fundamental questions that remain, particularly regarding how the SNAREs and Syt1 trigger fast Ca^2+^‐induced membrane fusion to enable the many wonders of the human brain.

## Conflict of interest

The authors declare no conflict of interest.
